# Zika viruses of African and Asian lineages cause fetal harm in a mouse model of vertical transmission

**DOI:** 10.1371/journal.pntd.0007343

**Published:** 2019-04-17

**Authors:** Anna S. Jaeger, Reyes A. Murrieta, Lea R. Goren, Chelsea M. Crooks, Ryan V. Moriarty, Andrea M. Weiler, Sierra Rybarczyk, Matthew R. Semler, Christopher Huffman, Andres Mejia, Heather A. Simmons, Michael Fritsch, Jorge E. Osorio, Jens C. Eickhoff, Shelby L. O’Connor, Gregory D. Ebel, Thomas C. Friedrich, Matthew T. Aliota

**Affiliations:** 1 Department of Veterinary and Biomedical Sciences, University of Minnesota, Twin Cities; St. Paul, MN, United States of America; 2 Arthropod-Borne and Infectious Diseases Laboratory, Department of Microbiology, Immunology and Pathology, Colorado State University; Ft. Collins, CO, United States of America; 3 Department of Pathobiological Sciences, University of Wisconsin-Madison; Madison, WI, United States of America; 4 Wisconsin National Primate Research Center, University of Wisconsin-Madison; Madison, WI, United States of America; 5 Department of Pathology and Laboratory Medicine, University of Wisconsin-Madison; Madison, WI, United States of America; 6 Department of Biostatistics and Medical Informatics, University of Wisconsin-Madison; Madison, WI, United States of America; CDC, UNITED STATES

## Abstract

Congenital Zika virus (ZIKV) infection was first linked to birth defects during the American outbreak in 2015/2016. It has been proposed that mutations unique to the Asian/American-genotype explain, at least in part, the ability of Asian/American ZIKV to cause congenital Zika syndrome (CZS). Recent studies identified mutations in ZIKV infecting humans that arose coincident with the outbreak in French Polynesia and were stably maintained during subsequent spread to the Americas. Here we show that African ZIKV can infect and harm fetuses and that the S139N substitution that has been associated with the American outbreak is not essential for fetal harm. Our findings, in a vertical transmission mouse model, suggest that ZIKV will remain a threat to pregnant women for the foreseeable future, including in Africa, Southeast Asia, and the Americas. Additional research is needed to better understand the risks associated with ZIKV infection during pregnancy, both in areas where the virus is newly endemic and where it has been circulating for decades.

## Author summary

Zika virus (ZIKV) was first discovered in Uganda in 1947, and is thought to have spread from Africa through equatorial Asia in the middle of the 20^th^ century. Along the way the virus diversified, so that now two genetic lineages, African and Asian/American, are recognized. Congenital Zika syndrome (CZS), the set of fetal and neonatal complications associated with ZIKV infection in pregnancy, was noted during the recent outbreak in the Americas. But the origins of CZS remain a mystery. In particular, it is unclear whether ZIKV recently acquired the ability to cause CZS, perhaps as Asian-lineage viruses spread to the Americas, or whether African-lineage viruses can also cause CZS. To address this question, we used a mouse model of vertical ZIKV transmission to assess pathogenic potential to the fetus of African and Asian/American ZIKV. Our data show that ZIKV of both African and Asian/American lineages can cause fetal harm in the mouse pregnancy model, and that this capacity does not require asparagine at amino acid residue 139, which recently emerged in Asian-lineage viruses and has been suggested to increase ZIKV’s pathogenic potential for fetuses. Our results, therefore imply that ZIKV infection during pregnancy poses a risk for fetal harm in all regions where the virus is endemic.

## Introduction

Zika virus causes adverse pregnancy outcomes including fetal loss, developmental abnormalities, and neurological damage, collectively termed congenital Zika syndrome (CZS) [1–4]. Why does CZS seem like a new complication when ZIKV has been circulating in Africa and Asia for decades? A provocative explanation for the recent appearance of CZS is that, during their geographic spread from Asia to the Americas, contemporary ZIKV strains acquired mutations that enhance neurovirulence and/or transplacental transmission. In several arboviruses, simple point mutations are known to result in changes in host range and/or the efficiency of infection and replication in key amplification hosts or vectors (see [5] for review). Accordingly, a single serine-to-asparagine substitution in the premembrane (prM) protein of ZIKV (S139N) that is unique to the Asian/American lineage viruses has been postulated to increase neurovirulence and contribute significantly to the microcephaly phenotype [6].

Yuan et al. [6] recently demonstrated that S139N substantially increased ZIKV infectivity in both human (*in vitro*) and mouse (*in vivo*) neural progenitor cells (NPCs), leading to restricted brain growth in an *ex vivo* embryonic mouse brain model, as well as higher mortality rates in neonatal mice following intracranial (i.c.) inoculation. Zhang et al. [7] reported similar findings, suggesting that American strains of ZIKV have a greater capacity for neurovirulence. However, accumulating data suggest that in historically endemic areas in Africa and Southeast Asia, ZIKV has always been teratogenic [8–12]. The degree to which the capacity to cause fetal harm is an emergent property unique to ZIKV circulating in the Americas, as well as the extent to which neurovirulence in laboratory models correlates with risk of fetal harm, remain open questions.

We aimed to better understand the hypothesized recent emergence of CZS by investigating whether both Asian- and African-lineage strains have the capacity to cause CZS. Using a vertical transmission model in mice, we assessed fetal outcomes after infection at embryonic day E7.5 by African- and Asian-lineage ZIKV strains, as well as the impact of the S139N substitution on the severity of gestational infection. We found that all ZIKV strains in our model caused adverse fetal outcomes, suggesting that the capacity of ZIKV to cause CZS does not map principally to polyprotein residue 139 and is not a newly acquired property.

## Methods

### Ethical approval

This study was approved by the University of Wisconsin-Madison and University of Minnesota, Twin Cities Institutional Animal Care and Use Committees (Animal Care and Use Protocol Numbers V5519 (UW) and 1804–35828 (UMN)).

### Cells and viruses

African Green Monkey kidney cells (Vero; ATCC #CCL-81) were maintained in Dulbecco’s modified Eagle medium (DMEM) supplemented with 10% fetal bovine serum (FBS; Hyclone, Logan, UT), 2 mM L-glutamine, 1.5 g/L sodium bicarbonate, 100 U/ml penicillin, 100 μg/ml of streptomycin, and incubated at 37°C in 5% CO_2_. *Aedes albopictus* mosquito cells (C6/36; ATCC #CRL-1660) were maintained in DMEM supplemented with 10% fetal bovine serum (FBS; Hyclone, Logan, UT), 2 mM L-glutamine, 1.5 g/L sodium bicarbonate, 100 U/ml penicillin, 100 μg/ml of streptomycin, and incubated at 28°C in 5% CO_2_. The cell lines were obtained from the American Type Culture Collection, were not further authenticated, and were not specifically tested for mycoplasma. ZIKV strain PRVABC59 (ZIKV-PR; GenBank:KU501215), originally isolated from a traveler to Puerto Rico in 2015 with three rounds of amplification on Vero cells, was obtained from Brandy Russell (CDC, Ft. Collins, CO). ZIKV-PR served as the backbone for the reverse genetic platform developed by Weger-Lucarelli et al. [13] upon which the single-amino acid substitution- N139S- was introduced. ZIKV strain DAK AR 41524 (ZIKV-DAK; GenBank:KX601166) was originally isolated from *Aedes luteocephalus* mosquitoes in Senegal in 1984, with a round of amplification on *Aedes pseudocutellaris* cells, followed by amplification on C6/36 cells, followed by two rounds of amplification on Vero cells, was obtained from BEI Resources (Manassas, VA). Virus stocks were prepared by inoculation onto a confluent monolayer of C6/36 mosquito cells. ZIKV strain FSS 13025 (ZIKV-CAM; GenBank:JN860885), originally isolated from a child in Cambodia in 2010 with three rounds of amplification on Vero cells, was obtained by Brandy Russell (CDC, Ft. Collins, CO). Virus stocks were prepared by inoculation onto a confluent monolayer of Vero cells.

### Generation of ZIKV prM mutant

An infectious clone for ZIKV-PR was constructed as previously described [13]. Infectious-clone derived virus (ZIKV-PR-IC) was recovered following electroporation of *in vitro* transcribed RNA into Vero cells. To engineer the N139S substitution into the ZIKV genome, the corresponding single-amino acid substitution was introduced into the ZIKV-PR-IC using the *in vitro* assembly cloning method [14]. The infectious clone plasmids were linearized by restriction endonuclease digestion, PCR purified, and ligated with T4 DNA ligase. From the assembled fragments, capped T7 RNA transcripts were generated, and the resulting RNA was electroporated into Vero cells. Infectious virus was harvested when 50–75% cytopathic effects were observed (6 days post transfection; ZIKV-N139S). Viral supernatant then was clarified by centrifugation and supplemented to a final concentration of 20% fetal bovine serum and 10 mM HEPES prior to freezing and storage as single use aliquots. Titer was measured by plaque assay on Vero cells as described in a subsequent section. We deep sequenced all of our challenge stocks (both wildtype and infectious clone-derived viruses) to verify the expected origin and amino acid at residue 139 (see details in a section below). All ZIKV stocks had the expected amino acid at residue 139: ZIKV-PR-IC (N), ZIKV-DAK (S), ZIKV-CAM (S), ZIKV-PR-N139S (S). Importantly, no single nucleotide polymorphisms were detected at residue 139 at a frequency greater than 1%, nor did we detect evidence of Dezidougou virus, an insect-specific *Negevirus* present in some ZIKV DAK AR 41524 stocks.

### Plaque assay

All ZIKV screens from mouse tissue and titrations for virus quantification from virus stocks were completed by plaque assay on Vero cell cultures. Duplicate wells were infected with 0.1 ml aliquots from serial 10-fold dilutions in growth media and virus was adsorbed for one hour. Following incubation, the inoculum was removed, and monolayers were overlaid with 3 ml containing a 1:1 mixture of 1.2% oxoid agar and 2X DMEM (Gibco, Carlsbad, CA) with 10% (vol/vol) FBS and 2% (vol/vol) penicillin/streptomycin. Cells were incubated at 37 °C in 5% CO_2_for four days for plaque development. Cell monolayers then were stained with 3 ml of overlay containing a 1:1 mixture of 1.2% oxoid agar and 2X DMEM with 2% (vol/vol) FBS, 2% (vol/vol) penicillin/streptomycin, and 0.33% neutral red (Gibco). Cells were incubated overnight at 37 °C and plaques were counted.

### Viral RNA isolation

Viral RNA was extracted from sera using the Viral Total Nucleic Acid Kit (Promega, Madison, WI) on a Maxwell 48 RSC instrument (Promega, Madison, WI). Viral RNA was isolated from homogenized tissues using the Maxwell 48 RSC Viral Total Nucleic Acid Purification Kit (Promega, Madison, WI) on a Maxwell 48 RSC instrument. Each tissue was homogenized using PBS supplemented with 20% FBS and penicillin/streptomycin and a tissue tearor variable speed homogenizer. Supernatant was clarified by centrifugation and the isolation was continued according to the Maxwell 48 RSC Viral Total Nucleic Acid Purification Kit protocol, and samples were eluted into 50 μl RNase free water. RNA was then quantified using quantitative RT-PCR. Viral load data from serum are expressed as vRNA copies/mL. Viral load data from tissues are expressed as vRNA copies/tissue.

### Quantitative reverse transcription PCR (QRT-PCR)

For ZIKV-PR, vRNA from serum and tissues was quantified by QRT-PCR using primers with a slight modification to those described by Lanciotti et al. to accommodate African lineage ZIKV sequences [15]. The modified primer sequences are: forward 5’-CGYTGCCCAACACAAGG-3’, reverse 5’-CACYAAYGTTCTTTTGCABACAT-3’, and probe 5’-6fam-AGCCTACCTTGAYAAGCARTCAGACACYCAA-BHQ1-3’. IUPAC nucleotide codes are as follows: Y: C or T; B: C or G or T; R: A or G. The RT-PCR was performed using the SuperScript III Platinum One-Step Quantitative RT-PCR system (Invitrogen, Carlsbad, CA) on a LightCycler 480 instrument (Roche Diagnostics, Indianapolis, IN). As a one-step assay, this assay uses the reverse primer to prime both reverse transcription and PCR and therefore detects positive-sense viral RNA. The primers and probe were used at final concentrations of 600 nm and 100 nm respectively, along with 150 ng random primers (Promega, Madison, WI). Cycling conditions were as follows: 37°C for 15 min, 50°C for 30 min and 95°C for 2 min, followed by 50 cycles of 95°C for 15 sec and 60°C for 1 min. Viral RNA concentration was determined by interpolation onto an internal standard curve composed of seven 10-fold serial dilutions of a synthetic ZIKV RNA fragment based on a ZIKV strain derived from French Polynesia that shares >99% similarity at the nucleotide level to the Puerto Rican strain used in the infections described in this manuscript.

### *In vitro* viral replication

Six-well plates containing confluent monolayers of Vero cells were infected with virus (ZIKV-PR-IC or ZIKV-PR-N139S), in triplicate, at a multiplicity of infection (MOI) of 0.01 PFU/cell. After one hour of adsorption at 37°C, the inoculum was removed and the cultures were washed three times. Fresh media were added and Vero cell cultures were incubated for 5 days at 37°C, with aliquots removed daily, diluted 1:10 in culture media, and stored at −80°C. Viral titers at each time point were determined by plaque titration on Vero cells and viral loads were determined by QRT-PCR.

### Mice

Female *Ifnar1-/-* mice on the C57BL/6 background were bred in the specific pathogen-free animal facilities of the University of Wisconsin-Madison Mouse Breeding Core within the School of Medicine and Public Health. Male C57BL/6 mice were purchased from Jackson Laboratories. Timed matings between female *Ifnar1-/-* mice and male C57BL/6 mice resulted in *Ifnar1-/+* progeny. Untimed, pregnant BALB/c mice were purchased from Charles River.

### Subcutaneous inoculation

All pregnant dams were between six and eight weeks of age. Littermates were randomly assigned to infected and control groups. Matings between female *Ifnar1*^*-/-*^ dams and wildtype sires were timed by checking for the presence of a vaginal plug, indicating a gestational age E0.5. At embryonic day 7.5 (E7.5), dams were inoculated in the left hind foot pad with 10^3^ PFU of ZIKV in 25 μl of sterile PBS or with 25 μl of sterile PBS alone to serve as experimental controls. All animals were closely monitored by laboratory staff for adverse reactions and signs of disease. A single sub-mandibular blood draw was performed 2 days post inoculation and serum was collected to verify viremia. Mice were humanely euthanized and necropsied at E14.5.

### Mouse necropsy

Following inoculation with ZIKV or PBS, mice were sacrificed at E14.5. Tissues were carefully dissected using sterile instruments that were changed between each mouse to minimize possible cross contamination. For all mice, each organ/neonate was evaluated grossly *in situ*, removed with sterile instruments, placed in a sterile culture dish, photographed, and further processed to assess viral burden and tissue distribution or banked for future assays. Briefly, uterus was first removed, photographed, and then dissected to remove each individual conceptus (i.e, fetus and placenta when possible). Fetuses and placentas were either collected in PBS supplemented with 20% FBS and penicillin/streptomycin (for plaque assays) or fixed in 4% PFA for imaging. Crown-rump length was measured by tracing distance from the crown of the head to the end of the tail using ImageJ. Infection-induced resorbed fetuses (~61%) were excluded from measurement analyses because they would not survive if the pregnancy was allowed to progress to term. We characterized an embryo as in the resorption process if it met the following criteria: significant growth retardation compared to litter mates and controls accompanied by clearly evident developmental delay, i.e., morphology was ill defined; or visualization of a macroscopic plaque in the uterus [16].

### Histology

Tissues were fixed in 4% paraformaldehyde for 24 hours and transferred into cold, sterile DPBS until alcohol processed and embedded in paraffin. Paraffin sections (5 μm) were stained with hematoxylin and eosin (H&E). Pathologists were blinded to gross pathological findings when tissue sections were evaluated microscopically. The degree of pathology at the maternal-fetal interface was rated on a scale of 0–4: 0 –no lesions (normal); 1 –mild changes; 2 –mild to moderate changes; 3 –moderate to severe changes; 4 –severe. The final scores were determined as a consensus score of three independent pathologists. Two patterns of injury were identified ranging from small focal or multifocal lesions to larger geographic areas of pathology. Each zone was assigned a quantitative score based on the following findings by each pathologist: 0—no lesions; 1 (mild) - 1–2 focal lesions or 5–10% of zone involved by pathology; 2 (mild to moderate) - 3–4 focal lesions or 10–15% of zone involved by pathology; 3 (moderate to severe) - 4–6 focal lesions or 15–25% of zone; and 4—greater than 6 focal lesions or >25% of zone involved by pathology. The majority of the placentas classified as severe (score of 4) usually had larger geographic areas of pathology exceeding 25%. The scoring of the 3 pathologists was within +/- 1 category for all placentas examined. For each zone in the placenta (myometrium, decidua, junctional zone, labyrinth, and chorionic plate/membranes) a ‘General’ overall score was determined, a score for the amount of ‘Inflammation’, and a score for direct ‘Vascular Injury’. The ‘General’ score was based on an interpretation of the overall histopathologic findings in each placenta, which included features of necrosis, infarction, hemorrhage, mineralization, vascular injury, and inflammation. The ‘Inflammation’ score quantified the amount of inflammation in that layer. The ‘Vascular Injury’ score assessed vascular wall injury (fibrinoid necrosis, endothelial swelling), dilatation of the vessels or spaces, and intraluminal thrombi. The myometrial layer representing the uterine wall and the chorionic plate/membranes were often not present in histologic sections and therefore meaningful comparisons between strains could not be assessed. The decidual layer (maternal in origin), the junctional zone composed of fetal giant cells and spongiotrophoblast, and the labyrinth layer (the critical layer for gas and nutrient exchange between the fetal and maternal vascular systems) were scored. Photomicrographs were obtained using a bright light microscope Olympus BX43 and Olympus BX46 (Olympus Inc., Center Valley, PA) with attached Olympus DP72 digital camera (Olympus Inc.) and Spot Flex 152 64 Mp camera (Spot Imaging), and captured using commercially available image-analysis software (cellSens DimensionR, Olympus Inc. and spot software 5.2).

### Intracranial inoculation

To test ZIKV strain neurovirulence, one-day-old BALB/c mice were intracranially (i.c.) inoculated at the lambda point with 10 PFU of virus or PBS alone. Each strain was tested in at least three litters. Following i.c. inoculation, mice were monitored twice daily for 28 days. Average survival time and percent mortality were calculated. Mice that succumbed within 24 hours of i.c. inoculation were excluded from further analyses. Mice that survived to 28 days were weighed and humanely euthanized. Survival curves are representative of two independent experiments, data were combined, and study site was included as a stratification factor in the analyses.

### Deep sequencing

Virus populations replicating in mouse sera were sequenced in duplicate using a method adapted from Quick et. al. [17]. Viral RNA was isolated from mouse sera using the Maxwell 16 Total Viral Nucleic Acid Purification kit, according to manufacturer’s protocol. Viral RNA then was subjected to RT-PCR using the SuperScript IV Reverse Transcriptase enzyme (Invitrogen, Carlsbad, CA). Input viral RNA was 10^6^ viral RNA templates per cDNA reaction. For sera from mice infected with ZIKV-PR-IC and ZIKV-PR-N139S, the cDNA was then split into two multi-plex PCR reactions using the PCR primers described in Quick et. al with the Q5 High-Fidelity DNA Polymerase enzyme (New England Biolabs, Inc., Ipswich, MA). For sera from mice infected with ZIKV-DAK, the cDNA was amplified in a PCR reaction for sequencing of a single amplicon with ZIKV-DAK specific primers (forward 5’-ACCTTGCTGCCATGTTGAGA-3’, reverse 5’CCGTACACAACCCAAGTCGA-3’) using Q5 High-Fidelity DNA Polymerase (New England Biolabs, Inc., Ipswich, MA). PCR products were tagged with the Illumina TruSeq Nano HT kit and sequenced with a 2 x 250 kit on an Illumina MiSeq.

A vial of the viral stocks used for primary challenge (ZIKV-PR-IC, ZIKV-PR-N139S, ZIKV-DAK, ZIKV-CAM), were each deep sequenced by preparing libraries of fragmented double-stranded cDNA using methods similar to those previously described [18]. Briefly, the sample was centrifuged at 5000 rcf for five minutes. The supernatant was then filtered through a 0.45-μm filter. Viral RNA was isolated using the QIAamp MinElute Virus Spin Kit (Qiagen, Germantown, MD), omitting carrier RNA. Eluted vRNA was then treated with DNAse I. Double-stranded DNA was prepared with the Superscript Double-Stranded cDNA Synthesis kit (Invitrogen, Carlsbad, CA) and priming with random hexamers. Agencourt Ampure XP beads (Beckman Coulter, Indianapolis, IN) were used to purify double-stranded DNA. The purified DNA was fragmented with the Nextera XT kit (Illumina, Madison, WI), tagged with Illumina-compatible primers, and then purified with Agencourt Ampure XP beads. Purified libraries were then sequenced with 2 x 300 bp kits on an Illumina MiSeq.

### Sequence analysis

Amplicon data were analyzed using a workflow we term “Zequencer 2017” (https://bitbucket.org/dhoconno/zequencer/src). Briefly, R1 and R2 fastq files from the paired-read Illumina miSeq dataset were merged, trimmed, and normalized using the bbtools package (http://jgi.doe.gov/data-and-tools/bbtools) and Seqtk (https://github.com/lh3/seqtk). Bbmerge.sh was used to merge reads, and to trim primer sequences by setting the forcetrimleft parameter 22. All other parameters were set to default values. These reads were then mapped to the reference amplicon sequences with BBmap.sh. Reads substantially shorter than the amplicon were filtered out by reformat.sh (the minlength parameter was set to the length of the amplicon minus 60). Seqtk was used to subsample to 1000 reads per amplicon. Quality trimming was performed on the fastq file of normalized reads by bbmap’s reformat.sh (qtrim parameter set to ‘lr’, all other parameters set to default). Novoalign (http://www.novocraft.com/products/novoalign/) was used to map each read to the appropriate ZIKV reference sequence: ZIKV-PRVABC59 KU501215, ZIKV DAK AR 41524 KX601166, ZIKV FSS13025 JN860885. Novoalign’s soft clipping feature was turned off by specifying the parameter “-o FullNW”. Approximate fragment length was set to 300bp, with a standard deviation of 50. We used Samtools to map, sort, and create an mpileup of our reads (http://samtools.sourceforge.net/). Samtools’ base alignment quality (BAQ) computation was turned off; otherwise, default settings were used. SNP calling was performed with VarScan’s mpileupcns function (http://varscan.sourceforge.net/). The minimum average quality was set to 30; otherwise, default settings were used. VCF files were annotated using SnpEff [19]. Accurate calling of end-of-read SNPs are a known weakness of current alignment algorithms [20]; in particular, Samtools’ BAQ computation feature is known to be a source of error when using VarScan (http://varscan.sourceforge.net/germline-calling.html). Therefore, both Novoalign’s soft clipping feature and Samtools’ BAQ were turned off to increase the accuracy of SNP calling for SNPs occurring at the end of a read.

Viral stock sequences were analyzed using a modified version of the viral-ngs workflow developed by the Broad Institute (http://viral-ngs.readthedocs.io/en/latest/description.html) implemented in DNANexus and using bbmap local alignment in Geneious Pro (Biomatters, Ltd., Auckland, New Zealand). Briefly, using the viral-ngs workflow, host-derived reads that map to a human sequence database and putative PCR duplicates were removed. The remaining reads were loaded into Geneious Pro and mapped to NCBI Genbank Zika virus reference sequences using bbmap local alignment. Mapped reads were aligned using Geneious global alignment and the consensus sequence was used for intra sample variant calling. Variants were called that fit the following conditions: have a minimum p-value of 10e-60, a minimum strand bias of 10e-5 when exceeding 65% bias, and were nonsynonymous.

### Data analysis

All analyses, except for deep sequencing analysis, were performed using GraphPad Prism. For survival analysis, Kaplan-Meier survival curves were analyzed by the stratified (by study site) log-rank test. Unpaired Student’s t-test was used to determine significant differences in crown-rump length, and viral loads of fetuses versus placentas. Fisher’s exact test was used to determine differences in rates of normal vs. abnormal concepti. One-way ANOVA with Tukey’s multiple comparison test was conducted to compare virus titers in maternal serum and to compare viral loads in placentas, fetuses, and concepti.

## Results and discussion

### Neurovirulence phenotypes of different ZIKV strains tested in neonatal mice

**Fig 1:**
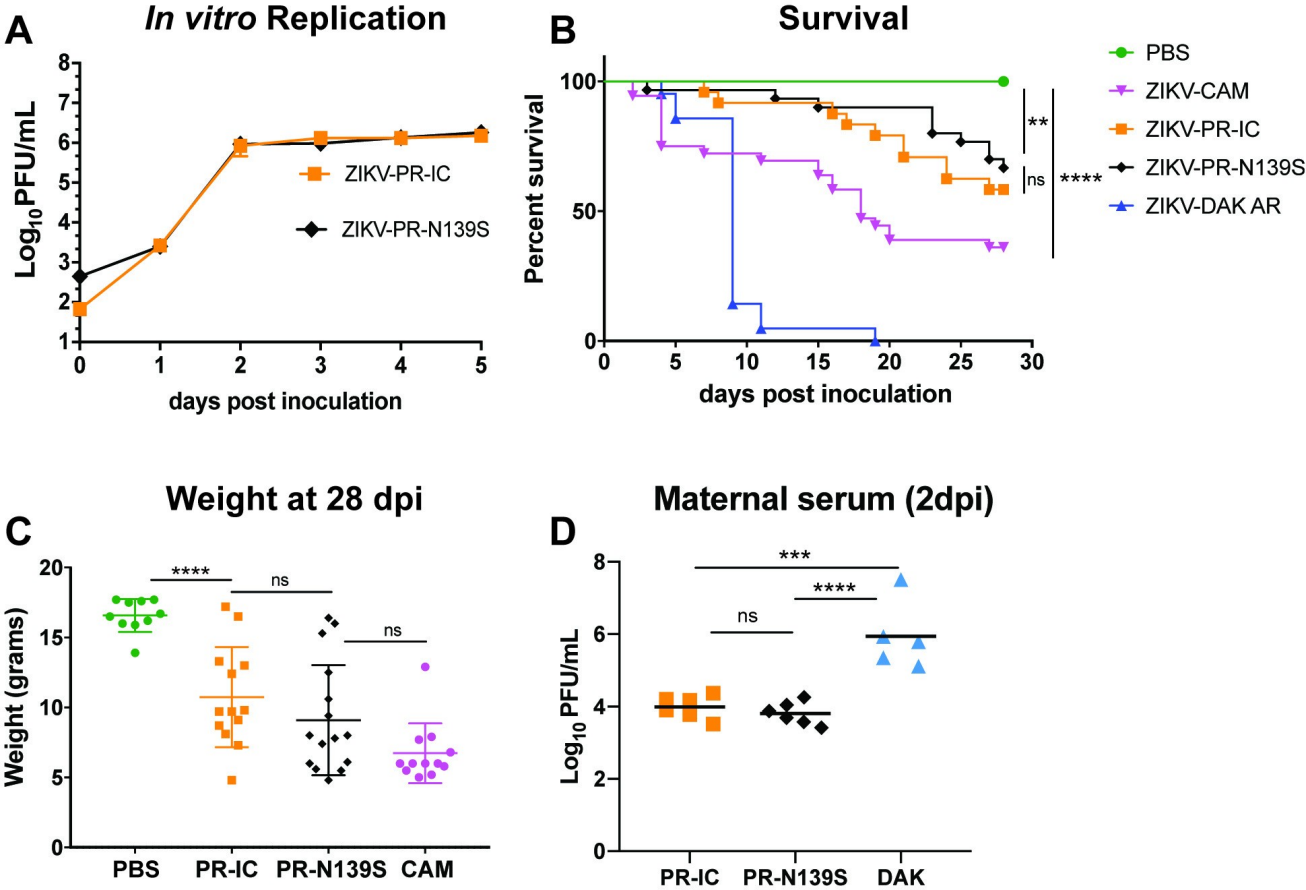
*In vitro* and *in vivo* characterization of ZIKV strains. **(a)** *In vitro* growth kinetics of ZIKV-PR-IC and mutant ZIKV-PR-N139S on Vero cells. Data points represent means of three replicates at each time point ± standard deviation. Cells were inoculated at an MOI of 0.01 PFU/cell. Titer was measured (PFU/ml) by plaque assay. Growth curves were not significantly different. **(b)** Survival curves of neonatal BALB/c mice intracranially inoculated with 10 PFU of different strains of ZIKV. PBS: n = 18; ZIKV-CAM: n = 36; ZIKV-PR-IC: n = 24; ZIKV-PR-N139S: n = 30; ZIKV-DAK: n = 20. All strains caused significant mortality by 28 dpi when compared to PBS (Fisher’s exact test). As compared to PBS controls: ****p < 0.001; ** *p* <0.002; ns, not significant. **(c)** Weight in grams of surviving intracranially inoculated pups at 28 days post infection. ****p < 0.0001; ns, not significant (student’s t-test). **(d)** Time-mated *Ifnar1-/-* dams were inoculated with 10^3^ PFU of ZIKV on E7.5 and maternal infection was confirmed by plaque assay on day 2 post inoculation. ***p < 0.002; **** *p* < 0.0001; ns, not significant (one-way ANOVA).

To characterize the range of pathogenic outcomes of congenital ZIKV infection and to assess the role of S139N on an alternate genetic background, we engineered the reverse amino acid substitution (asparagine reverted to serine at residue 139 in the viral polyprotein) into the Puerto Rican ZIKV isolate PRVABC59 (ZIKV-PR-N139S). Prior to use in mice, we assessed viral infectivity and replication of ZIKV-PR-N139S *in vitro* using Vero cells. ZIKV-PR-N139S and a control virus derived from an infectious clone bearing the wild type ZIKV-PRVABC59 consensus sequence (ZIKV-PR-IC; N at residue 139) gave similar growth curves (Fig 1A). These results suggest that the “reverse substitution” N139S did not have a significant effect on either infectivity or replicative capacity *in vitro*. Next, to assess whether N139S, in the context of the PRVABC59 genome, decreased mortality in the neonatal mouse model, we inoculated one-day-old BALB/c mice i.c. with 10 PFU of either ZIKV-PR-IC; ZIKV-PR-N139S; a ZIKV strain isolated in Cambodia in 2010 (ZIKV-CAM; FSS 13025; S at residue 139); a low-passage African ZIKV strain isolated in Senegal in 1984 (ZIKV-DAK; DAK AR 41524; S at residue 139); or, as a control, phosphate-buffered saline (PBS). Surprisingly, and in contrast to the results described by Yuan et al., i.c. inoculation of ZIKV-DAK and ZIKV-CAM resulted in 100% and 64% mortality, respectively, whereas 42% of mice succumbed to ZIKV-PR-IC and 33% to ZIKV-PR-N139S by 28 days post inoculation (dpi; Fig 1B). ZIKV-PR-IC and ZIKV-PR-N139S survival curves did not significantly differ (stratified log-rank test *p*-value = 0.1753), and all strains caused significant mortality (Fisher’s exact test) by 28 dpi as compared to the PBS-inoculated controls (ZIKV-CAM, ZIKV-DAK:*p*-value < 0.0001; ZIKV-PR-IC: *p*-value = 0.002; ZIKV-PR-N139S: *p*-value = 0.018). Additionally, pups from each treatment group that survived to 28 dpi were weighed to assess evidence for growth restriction (Fig 1C). Weight followed parallel trends observed with mortality, and pups in all ZIKV groups were too small to be weaned from dams at the standard 21-day timepoint. Weights from all ZIKV groups were significantly lower (Student’s t-test) than the PBS group (ZIKV-PR-IC: *p-*value < 0.0001, *t*-value = 4.93, df = 21; ZIKV-PR-N139S: *p-*value < 0.0001, *t*-value = 5.81, df = 24; ZIKV-CAM: *p-*value < 0.0001, *t*-value = 12.97, df = 20), ZIKV-PR-IC and ZIKV-PR-N139S did not differ significantly (*p*-value = 0.252, *t-*value = 1.17, df = 27), and the weights of surviving ZIKV-CAM pups were significantly lower than ZIKV-PR-IC (*p-*value < 0.0027, *t*-value = 3.36, df = 23) but not ZIKV-PR-N139S (*p-*value = 0.073, *t*-value = 1.87, df = 26). No ZIKV-DAK pups survived to 28 days to be included in these analyses. It is possible that the differences in survival in our study relative to Yuan et al. may be due to the use of a specific strain (PRVABC59 vs. GZ01). That is, the impact of a single amino acid substitution like S139N may vary in different strain backgrounds. However, these results are consistent with other studies in pregnant animal models that have provided evidence of neurovirulence and fetal demise caused by ZIKV strains isolated before the American outbreak [21–24]. Another possible explanation for the disparity in outcomes with ZIKV-CAM relative to other studies may be that previous studies only tested mutations at codon 139 in isolation on an otherwise isogenic background [6]. Likewise, others have used clone-derived viruses to understand overall ZIKV-CAM (strain FSS13025) pathogenesis [7,25,26]. Instead we directly compared pathogenic potential of a natural isolate of ZIKV-CAM. For some RNA viruses, swarm diversity can impact pathogenesis through cooperative interactions in the viral population [27] and this may not occur with clone-derived viruses or to the same degree as natural isolates.

### Maternal infection with African- and Asian-lineage ZIKV

The previous experiments establish the ability of each ZIKV strain tested to cause lethal infections in neonates, but direct intracranial inoculation does not fully recapitulate the events of a natural congenital infection. To better compare the abilities of these ZIKV strains to induce birth defects following vertical transmission, we used a previously established murine pregnancy model for ZIKV [22,28], in which dams lacking type I interferon signaling (*Ifnar1*^-/-^) were crossed with wildtype sires to produce heterozygous offspring. Because they have one intact *Ifnar1* allele, these offspring more closely resemble the immune status of human fetuses. Time-mated dams were inoculated subcutaneously in the footpad with 10^3^ PFU of ZIKV-PR-IC, ZIKV-PR-N139S, or ZIKV-DAK on E7.5, corresponding to the mid-to-late first trimester in humans [29]. We omitted ZIKV-CAM in the vertical transmission experiment due to previous experiments demonstrating its ability to cause fetal harm in *Ifnar1-/-* [22]. However, it should be noted that those experiments used intravaginal inoculation of ZIKV-CAM to examine the effects of gestational ZIKV infection, which precludes direct, quantitative comparisons to the results we describe here using subcutaneous inoculations. We collected serum samples from dams at 2 dpi to confirm infection and to sequence viral populations replicating *in vivo*. All dams were productively infected and one-way ANOVA with Tukey’s multiple comparisons test was conducted to compare titer between treatment groups. Maternal viremia was not significantly different in ZIKV-PR-IC- and ZIKV-PR-N139S-inoculated animals (*p-*value = 0.845), whereas ZIKV-DAK replicated to significantly higher titers on 2 dpi (ZIKV-PR-IC vs. ZIKV-DAK: *p-*value = 0.0002; ZIKV-PR-N139S vs. ZIKV-DAK: *p*-value < 0.0001). Deep sequencing of virus populations replicating in maternal serum confirmed that the N139S mutation was stably maintained *in vivo* (Table 1). Dams were monitored daily for clinical signs until time of necropsy. Overt clinical signs were only evident in ZIKV-DAK-inoculated dams and included hunched posture, ruffled fur, and hind limb paralysis indicative of neurotropism. All ZIKV-DAK-infected dams met euthanasia criteria at time of necropsy on E14.5.

**Table 1:** Deep sequencing of virus populations replicating in maternal serum. Serum samples from pregnant mice infected with ZIKV-PR-IC, ZIKV-PR-N139S, and ZIKV-DAK were deep sequenced in duplicate (R1, R2) and analyzed with the “Zequencer 2017” workflow to confirm the expected residue at position 139. The major codon found at residue 139 for each sample is indicated by gray shading.

												Major codon frequency at residue 139 (%)	Subsampled coverage depth at residue 139 (# reads)
**ZIKV-PRVABC59 (KU501215)**	130										140	**R1/R2**	**R1/R2**
	**S**	**A**	**Y**	**Y**	**M**	**Y**	**L**	**D**	**R**	**N**	**D**		
ZIKV-PR-IC-1	**·**	**·**	**·**	**·**	**·**	**·**	**·**	**·**	**·**	**·**	**·**	98.9/98.7	2000/149
ZIKV-PR-IC-2	**·**	**·**	**·**	**·**	**·**	**·**	**·**	**·**	**·**	**·**	**·**	99.2/94.5	2000/110
ZIKV-PR-IC-3	**·**	**·**	**·**	**·**	**·**	**·**	**·**	**·**	**·**	**·**	**·**	97.1/100	1999/16
ZIKV-PR-IC-4	**·**	**·**	**·**	**·**	**·**	**·**	**·**	**·**	**·**	**·**	**·**	99.8/99.3	2000/1023
ZIKV-PR-IC-5	**·**	**·**	**·**	**·**	**·**	**·**	**·**	**·**	**·**	**·**	**·**	99.8/99.9	2000/2000
ZIKV-PR-IC-6	**·**	**·**	**·**	**·**	**·**	**·**	**·**	**·**	**·**	**·**	**·**	99.9/99.7	2000/1715
ZIKV-PR-N139S-1	**·**	**·**	**·**	**·**	**·**	**·**	**·**	**·**	**·**	**S**	**·**	99.8/99.7	2000/1502
ZIKV-PR-N139S-2	**·**	**·**	**·**	**·**	**·**	**·**	**·**	**·**	**·**	**S**	**·**	99.9/99.7	2000/902
ZIKV-PR-N139S-3	**·**	**·**	**·**	**·**	**·**	**·**	**·**	**·**	**·**	**S**	**·**	100/99.8	1440/2000
ZIKV-PR-N139S-4	**·**	**·**	**·**	**·**	**·**	**·**	**·**	**·**	**·**	**S**	**·**	99.7/99.7	2000/2000
ZIKV-PR-N139S-5	**·**	**·**	**·**	**·**	**·**	**·**	**·**	**·**	**·**	**S**	**·**	99.6/98.9	2000/709
ZIKV-PR-N139S-6	**·**	**·**	**·**	**·**	**·**	**·**	**·**	**·**	**·**	**S**	**·**	99.6/99.7	2000/1585
**ZIKV-DAK AR 41524 (KX601166.2)**	130										140	**R1/R2**	**R1/R2**
	**S**	**A**	**Y**	**Y**	**M**	**Y**	**L**	**D**	**R**	**S**	**D**		
ZIKV-DAK-1	**·**	**·**	**·**	**·**	**·**	**·**	**·**	**·**	**·**	**·**	**·**	100/100	2000/2000
ZIKV-DAK-2	**·**	**·**	**·**	**·**	**·**	**·**	**·**	**·**	**·**	**·**	**·**	100/100	2000/2000
ZIKV-DAK-3	**·**	**·**	**·**	**·**	**·**	**·**	**·**	**·**	**·**	**·**	**·**	100/99.9	2000/2000
ZIKV-DAK-4	**·**	**·**	**·**	**·**	**·**	**·**	**·**	**·**	**·**	**·**	**·**	99.9/99.9	2000/2000
ZIKV-DAK-5	**·**	**·**	**·**	**·**	**·**	**·**	**·**	**·**	**·**	**·**	**·**	99.9/100	2000/2000

### Fetal outcomes after gestational infection with African- and Asian-lineage ZIKV

**Fig 2:**
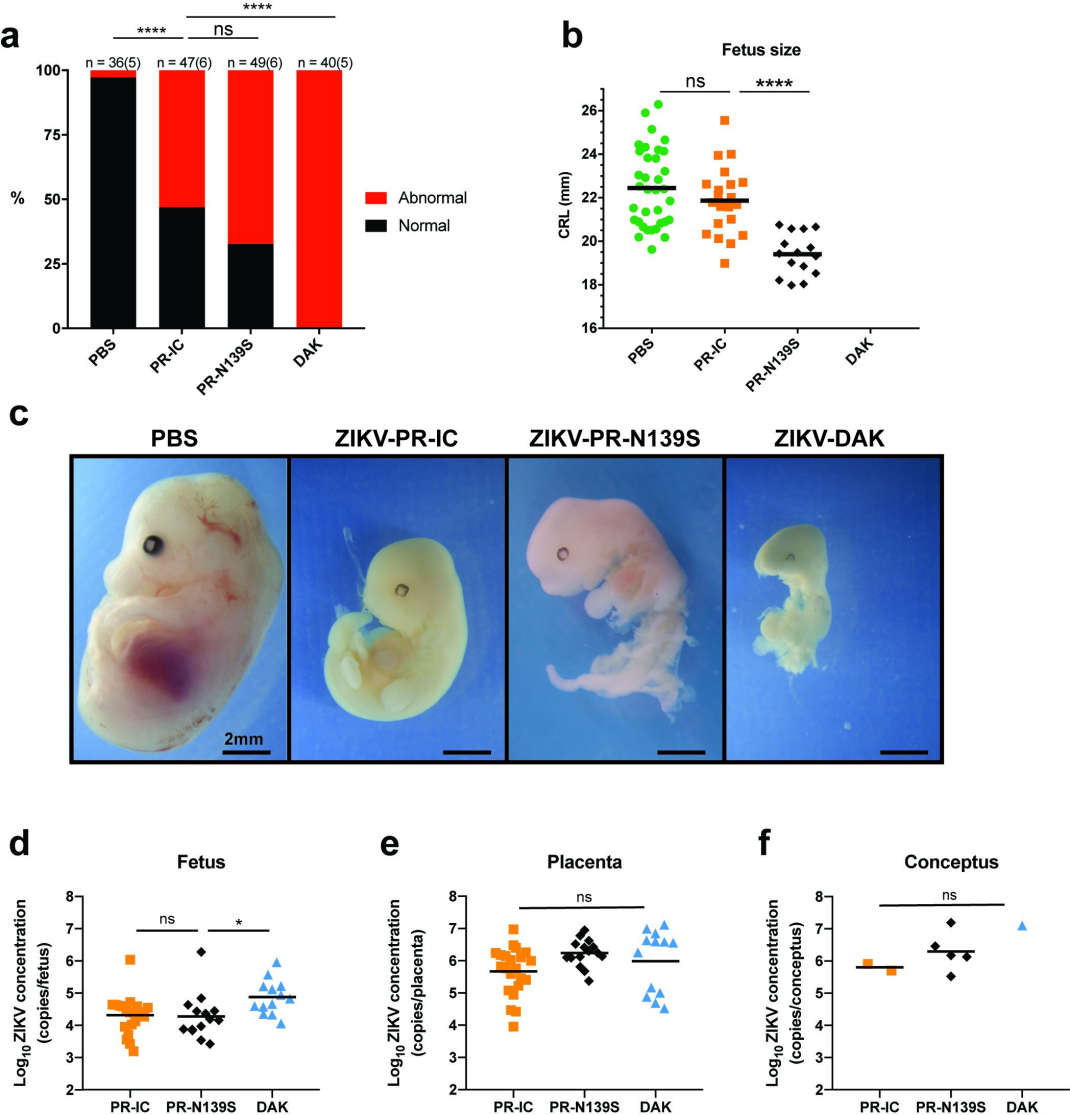
Fetal outcomes after maternal infection with ZIKV strains. **(a)** Rate of grossly normal (black) versus abnormal (red) fetuses at E14.5 after maternal infection at E7.5. An abnormal fetus was defined as resorption-prone. Data presented are for individual fetuses from 5–6 litters per treatment group. The n for each group is indicated above each bar. ****p<0.0001; ns, not significant (Fisher’s exact test). **(b)** Fetus size as assessed by crown-rump length (CRL) in mm using ImageJ software. CRL was only measured for fetuses determined to be grossly normal at E14.5. ****p<0.0001; ns, not significant (unpaired Student’s t-test). **(c)** Representative images of fetuses on E14.5 from each treatment group. Scale bar, 2 mm. PBS characterized as normal. ZIKV-PR-IC, ZIKV-PR-N139S, ZIKV-DAK characterized as abnormal. **(d-f)** Viral burdens were measured by qRT-PCR assay from individual homogenized placentas **(d)**, fetuses **(e)**, and concepti (when the fetus and placenta could not be separated due to severe resorption). **(f)** Symbols represent individual placenta, fetus, or conceptus from 3–5 independent experiments for each treatment group. Bars represent the mean viral burden of each treatment group. *p<0.05; ns, not significant (one-way ANOVA).

Next, to assess fetal outcomes, ZIKV-inoculated dams were sacrificed at E14.5. Gross examination of each conceptus (both fetus and placenta, when possible) revealed overt differences among fetuses within pregnancies and with uninfected counterparts. In general, fetuses appeared either grossly normal or abnormal, defined as being prone for embryo resorption (Fig 2A–2C) [16]. At time of necropsy, we observed high rates of resorption in both ZIKV-PR-IC- and ZIKV-PR-N139S-infected pregnancies. The proportion of abnormal fetuses for the two strains did not differ significantly (53.2% vs. 67.3%, Fisher’s exact test *p*-value = 0.21). In contrast, ZIKV-DAK-infected pregnancies resulted in 100% resorption of fetuses (Fig 2A). Only fetuses that appeared grossly normal were included for measurement of crown-rump length (CRL) to provide evidence for intrauterine growth restriction (IUGR). There was a modest reduction in size in grossly normal ZIKV-PR-IC fetuses. Mean CRL did not differ significantly (Student’s t-test) between fetuses of ZIKV-PR-IC- or PBS-inoculated dams (*p*-value = 0.22, *t-*value = 1.23, df = 54), whereas there was a statistically significant (Student’s t-test) reduction in mean CRL between fetuses whose dams were inoculated with ZIKV-PR-IC vs. ZIKV-PR-N139S (*p*-value < 0.0001, *t*-value = 5.42, df = 34; Fig 2B). This lack of apparent IUGR for ZIKV-PR-IC is contrary to other studies using Asian-lineage ZIKVs in which fetuses developed severe IUGR [22,28,30]. Again, this disparity may be the result of differences in timing of challenge and necropsy [31], subtle phenotypic differences in virus strain, dose and/or route of inoculation, or metrics for defining grossly normal fetuses compared to those undergoing resorption at a later embryonic age. Critically, the rates of resorption between ZIKV-PR-IC and ZIKV-PR-N139S were not significantly different and are consistent with the rates reported by Miner et al. [28]. These data also are consistent with a recent report suggesting that fetal demise may be a more common outcome of ZIKV infection than previously recognized [24]. Because our experiments only investigated the S139N mutation on the ZIKV-PRVABC59 background we cannot exclude the possibility that other SNPs, singly or in combination, may play functionally redundant roles with S139N in promoting neurovirulence. It is important to note that all studies seeking to link viral genotype to phenotype using reverse genetics can be limited by the genetic context of the cloned virus(es) chosen for analysis. In our case, we do not observe a significant difference in pathogenic potential of ZIKV encoding serine or asparagine at polyprotein position 139. But we examined this particular point mutation in a single genetic background. It is possible that S139N may act in concert with other polymorphisms to affect ZIKV neurotropism, neurovirulence, and/or other factors, such that S139N mutations examined in the context of different ZIKV strains might have different phenotypic effects. Nonetheless, our results, together with others, clearly show that ZIKV of multiple genetic lineages can cause fetal harm irrespective of the amino acid residue present at position 139.

### Confirmation of vertical transmission

To confirm vertical transmission of ZIKV to the developing conceptus, viral loads were measured from representative placentas and fetuses from each litter of all treatment groups by quantitative RT-PCR (Fig 2D–2F). vRNA was detected in all fetuses and placentas that were tested. Viral loads were significantly higher (Student’s t-test) in placentas than in fetuses (*p*-value < 0.0001, *t*-value = 5.04, df = 95; Fig 2D and 2E), whereas placental viral loads were not significantly different between groups infected with different viruses nor among littermates within the same litter (ZIKV-PR-IC vs. ZIKV-PR-N139S: *p*-value = 0.063, df = 47; ZIKV-PR-IC vs. ZIKV-DAK: *p*-value = 0.43, df = 47; ZIKV-PR-N139S vs. ZIKV-DAK: *p*-value = 0.64, df = 47; one-way ANOVA with Tukey’s multiple comparisons). In contrast, ZIKV-DAK infected fetuses had significantly higher viral loads when compared to ZIKV-PR-IC and ZIKV-PR-N139S (ZIKV-PR-IC vs. ZIKV-DAK: *p*-value = 0.04, df = 44; ZIKV-PR-N139S vs. ZIKV-DAK: *p*-value = 0.04, df = 44; one-way ANOVA with Tukey’s multiple comparisons). Although day 2 viremia was significantly higher in ZIKV-DAK-infected dams, the fact that fetal and placental viral loads are broadly similar, both within and across treatment groups indicates that it is not simply a matter of enhanced replication of the African virus in the dam causing more severe fetal outcomes. Still, we cannot exclude the possibility that the more severe fetal outcomes observed with the African virus are the result of IFNAR-dependent fetal demise [22] singly or in combination with IFNAR-independent causes of birth defects like poor maternal health, direct pathogenic effects of the virus infection (as indicated by a significant, albeit moderate, increase in fetus viral loads), or a bystander effect associated with immune responses unrelated to type 1 interferons. Additionally, comparable viral loads were measured from both grossly normal and abnormal fetuses and placentas. Detection of ZIKV RNA in grossly normal fetuses does not preclude the possibility that pathology may develop later in pregnancy or even postnatally, consistent with reports from humans that the effects of *in utero* exposure may not be evident at birth [32].

### Maternal and fetal histopathology analysis

**Fig 3:**
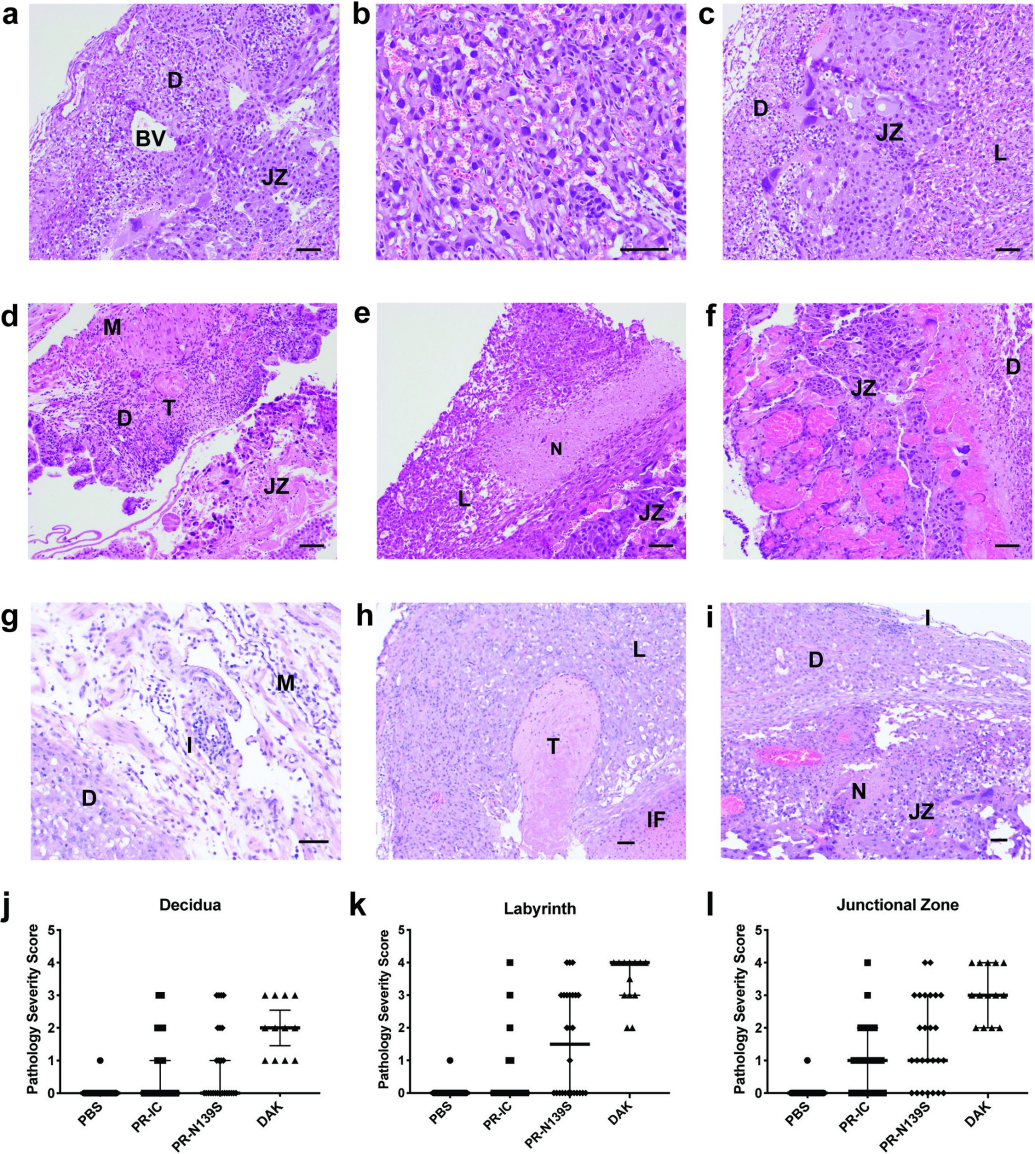
Placenta histopathology analysis: Hematoxylin and eosin (H&E) staining of placenta and fetus. **(a-c)** Normal histologic features of each placental zone (decidual layer (D), labyrinth layer (L), and junctional zone (JZ)) from concepti from dams inoculated with PBS. BV, normal decidual blood vessels. **(d-f)** Severe histopathologic injury patterns for each zone from placenta from ZIKV-inoculated dams. **(d)** Myometrium (M) and decidua (D) from a ZIKV-PR-IC placenta with increased inflammation, multiple thrombi (T) in the decidua, and a necrotic JZ. **(e)** L from a ZIKV-DAK placenta with focal necrosis (N), lack of blood in most vascular spaces, and numerous degenerating cells. **(f)** JZ from a ZIKV-DAK placenta with markedly dilated blood vessels, focal thrombi, and a layer of necrosis at the interface with the decidua. **(g)** D and M from a ZIKV-PR-N139S placenta with inflammation (I). **(h)** L from a ZIKV-PR-N139S placenta with T and infarction (IF). **(i)** JZ from a ZIKV-PR-IC with N and I. **(j-l)** The degree of placental pathology was rated on a scale of 0–4: zero represents normal histologic features and 4 represents the most severe features observed. Each zone of the placenta was scored individually for general overall pathology, amount of inflammation, and amount of vascular injury with a consensus score for each placenta derived from three independent pathologists. Only ‘General’ scores are shown because they were representative of the ‘inflammation’ and ‘vascular injury’ categories and do not differ significantly from ‘general’. Error bars represent 95% confidence interval from the median. Data are representative of 3–5 independent experiments for each treatment group. Scale bar, 50 μm.

To better understand the impact of *in utero* ZIKV infection, tissues of the developing placenta and decidua were evaluated microscopically. In PBS-inoculated dams, we observed normal decidua, junctional zone, and labyrinth with normal maternal and fetal blood spaces (Fig 3A–3C). In contrast, ZIKV-inoculated dams displayed varying degrees of placental pathology, including vascular injury involving maternal and/or fetal vascular spaces, infarction (obstructed blood flow), necrosis, inflammation, and hemorrhage (Fig 3D–3F). There also were clear strain-specific differences in the amount of placental pathology, with ZIKV-DAK displaying the most severe histologic phenotype, consistent with gross observations (Fig 3G–3I). These data are consistent with some reports suggesting that African-lineage ZIKVs might have greater systemic virulence in mice than Asian ones (reviewed in [33]). The underlying mechanism responsible for enhanced virulence remains unknown, but it is important to note that many of the available African isolates have undergone passage in suckling mouse brain, whereas ZIKV-DAK used in our experiments has not. Variations in passage history could result in small, but biologically important, genotypic and phenotypic differences. Still, reports with low-passage African isolates also suggest that these African strains display increased virulence in numerous mouse models when compared to Asian isolates (e.g., [34–36]). As a result, maternal illness could be a potential confounder and the phenotype reported here may not necessarily reflect a pregnancy-specific effect. Ultimately, it will be important to determine whether these results can be recapitulated in a translational model that provides a closer representation of the human morphological, developmental, and immune environment at the maternal-fetal interface, e.g., macaque monkeys.

Importantly, we assessed outcomes following only a single infection time point at E7.5 and outcomes may differ depending on the timing of infection. Critically, the placenta acts as a barrier against infections but does not have a definitive structure in mice until the midpoint of gestation: ~E10.5–11.5 [37]. It is possible then that inoculations later during gestation, when there is a more fully developed placental barrier, may exhibit greater resistance to infection and result in less severe pathologic outcomes [38].

## Conclusions

Together our data show that infection with ZIKV isolates of either the African or Asian lineages during pregnancy can lead to fetal harm, with varying levels of damage to maternal, placental, and fetal tissues, frequently including death of the developing fetus. Likewise, intracranial inoculation of neonatal mice confirmed a similar neurovirulence phenotype across ZIKV lineages. A recent study identified the potential capacity of an ancestral Asian strain to cause fetal brain infection after maternal infection in mice [39]; that study used a cloned virus created from reverse genetics that corresponded to a publicly available consensus ZIKV genome sequence. To our knowledge, our study is the first to assess a low-passage African isolate, or the S139N substitution, in a vertical transmission model. The observation that a low-passage African ZIKV isolate can cause severe fetal harm suggests that, for decades, ZIKV could have been causing pregnancy loss and birth defects, which were either undiagnosed or attributed to other causes. If this hypothesis is correct, CZS is not a new syndrome caused by a recently emerged ZIKV variant, but rather an old entity that was only recognized in the large-scale American ZIKV outbreak that began in 2014–15. These results provide compelling motivation to re-evaluate hypotheses explaining the emergence of CZS. A lack of thorough surveillance, together with myriad co-circulating pathogens causing febrile illnesses, make understanding both the past and current prevalence of gestational ZIKV infection and any resulting fetal outcomes in Africa challenging [40–42]. Recent seroprevalence studies have now identified low, but consistent circulation of ZIKV in several African [43–46] and southeast Asian [47] countries, indicating a large population potentially at risk. Accurate assessment of the risk posed by ZIKV infection to pregnant women and their babies in both Africa and southeast Asia should be a priority.
